# Intellectual Disability in Children with Attention Deficit Hyperactivity Disorder

**DOI:** 10.1016/j.jpeds.2013.02.043

**Published:** 2013-09

**Authors:** Alka Ahuja, Joanna Martin, Kate Langley, Anita Thapar

**Affiliations:** 1 Ty Bryn Unit, St Cadocs Hospital, Newport, United Kingdom; 2 Child and Adolescent Psychiatry Section, Institute of Psychological Medicine and Clinical Neurosciences, Cardiff University School of Medicine, Cardiff, United Kingdom; 3 Medical Research Council Center for Neuropsychiatric Genetics and Genomics, Cardiff University School of Medicine, Cardiff, United Kingdom

**Keywords:** ADHD, Attention deficit hyperactivity disorder, ALSPAC, Avon Longitudinal Study of Parents and Children, ASD, Autism spectrum disorder, CAPA, Child and Adolescent Psychiatry Assessment, CD, Conduct disorder, CNV, Copy number variant, DSM-III-R, *Diagnostic and Statistical Manual of Mental Disorders, 3rd edition revised*, DSM-IV, *Diagnostic and Statistical Manual of Mental Disorders, 4th edition*, ID, Intellectual disability, ODD, Oppositional defiant disorder

## Abstract

**Objective:**

To determine whether children with attention deficit hyperactivity disorder (ADHD) and mild intellectual disability (ID) are a clinically distinct ADHD subgroup.

**Study design:**

This was a cross-sectional study comparing clinical characteristics (ADHD subtypes, total number of symptoms, and rates of common comorbidities) between children with ADHD and mild ID and those with ADHD and IQ test scores >70, and also between children with ADHD and ID and a general population sample of children with ID alone. The sample comprised a clinical sample of children with ADHD with ID (n = 97) and without ID (n = 874) and a general population sample of children with ID and without ADHD (n = 58).

**Results:**

After correcting for multiple statistical tests, no differences were found between the 2 ADHD groups on any measure except the presence of conduct disorder (CD) symptoms and diagnoses. Children with ADHD and ID had higher rates of both (OR, 2.38; 95% CI, 1.71-3.32 and OR, 2.69; 95% CI, 1.69-4.28, respectively). Furthermore, children with ADHD and ID had significantly higher rates of oppositional defiant disorder (OR, 5.54; 95% CI, 2.86-10.75) and CD (OR, 13.66; 95% CI, 3.25-57.42) symptoms and a higher incidence of oppositional defiant disorder diagnoses (OR, 30.99; 95% CI, 6.38-150.39) compared with children with ID without ADHD.

**Conclusion:**

Children with ADHD and mild ID appear to be clinically typical of children with ADHD except for more conduct problems. This finding has implications for clinicians treating these children in terms of acknowledging the presence and impact of ADHD symptoms above and beyond ID and dealing with a comorbid CD.

Attention deficit hyperactivity disorder (ADHD) is a disabling condition, affecting 1.4%-6% of children.^1^ Little is known of the clinical presentation and etiology of ADHD in children with intellectual disability (ID), because those with lower cognitive ability (IQ scores <70) are often excluded from studies of ADHD,^2^ despite evidence that ADHD is more common in children with ID, and that the risk increases with increasing severity of ID.^3^

It has been suggested that ADHD does not occur in children with ID, and that any inappropriate behavior in children with ID is secondary to “mental impairment.”^4^ That view is not supported by current evidence, however. Studies have shown that ADHD occurs more commonly in these children but may be underdiagnosed owing to such issues as “diagnostic overshadowing,” the tendency of clinicians to overlook additional psychiatric diagnoses after a diagnosis of ID is made, or “masking,” in which the clinical characteristics of a mental disorder are masked by a cognitive, language, or speech deficit.^5^

A population-based study estimating the prevalence of psychiatric diagnoses in children with ID identified hyperkinetic disorder as the most common psychiatric disorder.^6^ Studies of children with mild and borderline ID have identified ADHD in 8%-39% of cases.^7-9^ A crucial clinical issue is whether or not the clinical pattern of comorbidity in this group is the same as that seen in children with ADHD but without ID. This is important in determining the level and type of services and clinical care required for this subgroup.

In the present study, we compared the rates of comorbid problems and ADHD symptom levels in 2 groups of children with ADHD, 1 group with ID (ADHD + ID group) and the other group without ID (ADHD-only group). Consistent with previous studies of ADHD, we defined ID is an IQ test score <70. We hypothesized that the ADHD profiles in the 2 groups (ADHD + ID [IQ <70] vs ADHD-only [IQ ≥70]) would be highly similar in terms of symptoms, rates of subtypes, and patterns of comorbid problems (ie, oppositional behaviors, conduct disorder [CD], anxiety, and depression).

## Methods

Participants were recruited from more than 30 child and adolescent mental health services or community pediatric outpatient clinics in Wales, England, and Scotland for a genetic study of ADHD. Given this study's focus on evaluating for the presence of nonsyndromal ID in children with ADHD, *International Statistical Classification of Diseases and Related Health Problems, 10th revision*^10^ and *Diagnostic and Statistical Manual of Mental Disorders, 4th edition* (DSM-IV)^11^ exclusion criteria were used. Children with a known diagnosis of schizophrenia, autism spectrum disorder (ASD), bipolar disorder, Tourette syndrome, epilepsy, brain damage, or any other neurologic or genetic disorder were excluded. Information on these conditions was derived from a questionnaire completed by the referring clinician, diagnostic interview information obtained from parents, and quality control of genetic data performed as part of the genetic study. Children with IQ <50 were also excluded, because the study focused on mild ID, and the assessment measures have not yet been validated in individuals with severe ID.

A total of 971 children met the inclusion criteria and had sufficient data for analysis. All of these children met the DSM-IV^11^ or *Diagnostic and Statistical Manual of Mental Disorders, 3rd edition revised* (DSM-III-R)^12^ criteria for a diagnosis of ADHD, which was confirmed through research diagnostic interviews.^13^ The children ranged in age from 5 to 17 years (mean age, 10.1 ± 2.8 years), and included 148 females (15.2%). The study received ethical approval from the North West England and Wales Multicentre Research Ethics Committees. For all subjects, written informed consent was obtained from parents and assent/consent from children.

Cognitive ability was assessed using *the Wechsler Intelligence Scale for Children versions III* (n = 381) and *IV* (n = 590)^14,15^ to obtain an estimate of full-scale IQ (using all required subtests). Two versions of this assessment tool were used because version IV was released during the study period. The assessment was performed by trained psychologists. In children who had recently undergone IQ assessment in school, that score was used to determine ID status. In accordance with *International Statistical Classification of Diseases and Related Health Problems, 10th revision* and DSM-IV criteria, children with an IQ score of 50-69 were considered to have mild mental retardation/ID and classified in the ADHD + ID group. Children with an IQ score ≥70 were classified in the ADHD-only group.

ADHD symptoms, impairment, and diagnoses were confirmed using the Child and Adolescent Psychiatry Assessment (CAPA),^13^ a research diagnostic interview with parents. Interviews were performed by trained psychologists supervised weekly by a child psychiatrist. Interrater reliability for ADHD was perfect (κ = 1.0). Information on ADHD symptoms and school impairments was obtained using the Child ADHD Teacher Telephone Interview,^16^ the DuPaul teacher rating scale,^17^ or the Conners teacher rating scale.^18^ A diagnosis of ADHD required that the child have symptoms meeting DSM-IV or DSM-III-R criteria, substantial impairment from symptoms at home, and pervasive symptoms and impairment in the school setting.

The CAPA was also used to assess current symptoms, impairment, and DSM-IV diagnoses of comorbid oppositional defiant disorder (ODD), CD, anxiety disorders (ie, generalized anxiety disorder, social anxiety, and separation anxiety), depression, and mania. Comorbid symptoms were also assessed using the child version of the CAPA^19^ for children aged ≥12 years. Comorbid anxiety or depression symptoms were endorsed if reported by the parent or child. Owing to the scarcity of anxiety and depression diagnoses in the sample, only symptoms of these disorders could be analyzed. Interrater reliability for parent-rated CD symptoms was very good (intraclass correlation, 0.98).

### Avon Longitudinal Study of Parents and Children

To compare clinical variables found to be associated in the primary analysis in the ADHD + ID and ADHD-only groups, we turned to the Avon Longitudinal Study of Parents and Children (ALSPAC), a large, well-characterized longitudinal dataset. Details of the study methodology are available elsewhere.^20^ Ethical approval for all aspects of the study was obtained from the ALSPAC Law and Ethics Committee and the local Research Ethics Committees. Parents provided written consent and the children provided assent at each assessment. IQ had been assessed at age 8 years using the *Wechsler Intelligence Scale for Children version III*.^14^ Children who scored between 50 and 69 on the IQ test and had no diagnosis of ADHD or ASD were included in our analysis. A total of 74 children (1.2% of the ALSPAC sample with complete data on these measures) met these criteria. Data on ADHD, ASD, ODD, and CD symptoms and diagnoses were collected from participants at age 128 months, using the parent and teacher Development and Well-Being Assessment.^21^ Complete clinical data were available for 58 children, who constituted the ID-only group. These children were 10-11 years old at the time of clinical assessment (mean, 10.8 ± 0.1 years), and 27 were female (46.6%).

### Statistical Analyses

The ADHD clinical sample was divided into those with ID (ADHD + ID; n = 97) and those without ID (ADHD-only; n = 874). The 2 groups were compared on each of the clinical factors identified. All descriptive statistics are presented as raw scores for ease of interpretation. Where a variable was nonnormally distributed, the scores were naturally logarithmically transformed, and analyses were run on transformed scores.

Clinical predictor variables were used to predict binary outcomes (ADHD + ID or ADHD-only) using regression analyses. All analyses included child's age at the time of assessment as a covariate. Sex was not included as a covariate, because it was not associated with the presence or absence of ID. Clinical variables were assessed both categorically and continuously, whenever relevant. All analyses were performed using SPSS version 16 (IBM, Armonk, New York). To take into account multiple testing, Bonferroni correction for the number of variables tested was used, α was set at *P* = .003 (0.05/15) for the 15 tests performed.

Based on our results, a hypothesis-driven comparison of the ADHD + ID and ID-only samples was performed for rates of diagnoses and symptom counts for ODD and CD, adjusted for the covariates age, sex, and IQ. CD items available in both datasets were summed and used to generate the CD diagnoses (lying, fighting, breaking curfew, stealing, truancy, running away from home, and bullying).

## Results

### Sample Description: Clinical ADHD Sample

At the time of assessment, 74.3% of the children (n = 721) met the criteria for DSM-IV ADHD Combined type, 6.0% (n = 58) met the criteria for DSM-IV ADHD Inattentive type, 9.6% (n = 93) met the criteria for DSM-IV ADHD Hyperactive-Impulsive type, and the remaining 10.2% (n = 99) met the criteria for DSM-III-R ADHD. The rates of comorbid disorders were 45.2% (n = 435) for DSM-IV ODD, 17.6% (n = 171) for DSM-IV CD, 6.1% (n = 59) for any anxiety disorder, and 1.1% (n = 11) for any depressive disorder. The mean IQ test scores were 61.8 ± 5.4 for the ADHD + ID group and 87.8 ± 11.4 for the ADHD-only group; the range of scores was 50-69 for the ADHD + ID group and 70-139 for the ADHD-only group. The scores were normally distributed for the sample as a whole.

### Sample Description: ID-Only ALSPAC Sample

The mean IQ score of the ID-only group was 64.8 ± 4.5. At the 128-month assessment, 2 of the children in the ID-only group met the criteria for DSM-IV ODD (3.4%), and none met the criteria for DSM-IV CD.

### Analysis of Clinical Features in Children with ADHD

The ADHD + ID group was older than the ADHD-only group, but the 2 groups did not differ in terms of sex distribution. Table I presents the descriptive statistics and results of regression analyses with age included as a covariate. Although a trend for the ADHD + ID group to be more likely to have the DSM-IV Combined ADHD subtype was seen, this result did not withstand correction for multiple testing. Otherwise, the 2 groups of children were similar in terms of ADHD subtypes and Inattentive, Hyperactive-Impulsive, and total ADHD symptoms. The 2 groups also had similar rates of ODD diagnoses and of anxiety and depression symptoms. There was a trend for children with ADHD + ID to have on more ODD symptoms on average. The ADHD + ID group had more symptoms of CD and were more likely to have a diagnosis of CD; these associations remained after multiple testing was taken into consideration.

### Analysis of Clinical Features in Children with ID-Only

Results of the comparison of ADHD + ID and ID-only groups (clinical sample vs population sample) are presented in Table II. The 2 groups differed significantly in terms of sex (more boys in the ADHD + ID group), but not in age at assessment. Although the range of IQ scores was similar in the 2 groups, the ADHD + ID group had significantly lower scores (OR, 0.88; 95% CI, 0.82-0.95; *P* = .001). After adjusting for sex, age, and IQ score, the children in the ADHD + ID group were significantly more likely to have a diagnosis of ODD, and had significantly more symptoms of ODD and CD. Statistical assessment of the between-group difference in the rate of CD diagnoses was not possible, given the rate of 0 in the ID-only group.

## Discussion

In our clinical sample of children with ADHD, those with and without mild ID (IQ score 50-69) exhibited similar patterns of ADHD subtypes, total number of ADHD symptoms, and comorbidity with ODD, anxiety, and depression. The children in the ADHD + ID group did have higher rates of CD symptoms and diagnoses, however. To explore these results further and test whether these differences were related to an increase in behavioral problems in children with ID in general, we compared our ADHD + ID group with a population-based sample of children with ID but without ADHD. We found significantly higher rates of ODD diagnoses and ODD and CD symptoms in the ADHD + ID group, suggesting that the combination of ADHD and ID results in increases in the rates of comorbid CD and ODD beyond the rates of these disorders in individuals with ID only or ADHD only. The mean IQ score was lower in the ADHD + ID group compared with the ID-only group. This difference in IQ may be related to selective attrition in the ALSPAC sample,^22^ clinical ascertainment effects, or the effects of ADHD on IQ test performance. Regardless, when we matched the groups on IQ scores by selecting a subsample of children with ADHD + ID (n = 58, to match the number of children in the ID-only group), we obtained the same pattern of results (Table III; available at www.jpeds.com).

ADHD is one of the most common forms of psychopathology in children with ID.^7^ Generally, children with ID are neglected in the medical field, perhaps in part because of their poor ability to communicate and social disadvantages.^2^ They are frequently excluded from clinical, etiologic, and treatment studies,^2^ and thus few studies to date have examined the clinical presentation, etiology, patterns of service use, and treatment of ADHD in children with ID. Moreover, there are few standardized norms or guidelines in the classification systems for identifying “normal” or “usual” amounts of inattention, overactivity and impulsivity in persons with ID.

Studies with clinic-referred children and community and population samples have shown consistently higher rates of ADHD in children with ID and higher rates of ID in children with ADHD.^23-25^ There has been less work on whether the clinical features and levels of ADHD symptomatology differ between children with ID and those without ID. One study found equivalent levels of ADHD symptoms in preschool-age children with ADHD and normal IQ and those with ADHD and ID.^26^ A population study assessed overactivity, inattention, and impulsivity symptoms using the Strengths and Difficulties Questionnaire in children with IQ <70 and those with IQ ≥70 and found no differences between the 2 groups.^24^ A recent longitudinal study compared children with ADHD with IQ <85 (indicating ID or borderline IQ) and those with IQ ≥85 and found similar inattentive and hyperactive-impulsive ADHD symptom trajectories over a 3-year period.^27^ An important difference noted in that sample was that the children with lower IQ tended to meet the diagnostic criteria for ADHD at an earlier age and to have more diagnostic stability than the children with higher IQ, indicating a more severely impairing form of ADHD. A study that addressed this question from the other direction found that although the children with developmental delay (IQ <85) had a higher rate of ADHD than the typically developing children (IQ ≥85), the pattern of ADHD subtypes and levels of inattentive and hyperactive-impulsive symptoms were similar in the 2 groups.^9^

The few studies examining whether rates of comorbid psychiatric disorders in ADHD differ in children with and without ID have shown mixed results. Some studies have found higher rates of comorbid impairments in social skills, conduct problems, aggression, and noncompliance in children with ADHD and ID compared with children with ADHD and normal IQ.^26,28^ In contrast, in community samples, the profiles of comorbidity with emotional and conduct problems in children with ADHD symptoms did not differ according to the presence of mild ID (IQ <70)^24^ or using a cutoff of IQ <85.^29^ A third study found higher rates of noncompliance, anxiety, depression, and social problems in those with ADHD and ID compared with those with ID alone.^30^

A limitation of previous research is that questionnaires generally have been used to assess comorbidity rather than the more in-depth standardized diagnostic assessment methods used in the present study. Another strength of the present study is the relatively large sample size of the ADHD group compared with previous studies. The IQ cutpoint used to delineate comparison groups in the literature varies, such that in some studies, children with borderline ID (IQ 70-85) are included in the group of children with mild ID. Although the present study focused on comparing children based on an IQ cutpoint of 70, reanalysis of the data after dividing the children into 3 groups (mild ID: IQ 50-69 [n = 97], borderline ID: IQ 70-84 [n = 380], and typically developing: IQ ≥85 [n = 494]) showed a similar pattern of results (Table IV; available at www.jpeds.com).

An important limitation of the present study is that the presence of ID was defined based primarily on IQ score, because no measure of adaptive functioning was available. Another limitation is that disharmonic IQ profiles were not considered, and thus children with significant performance IQ and verbal IQ discrepancies might not necessarily be considered to have ID in clinical practice. However, given that IQ alone is often the basis for exclusion criteria in ADHD research, the implications of our findings are valid in this context. Samples are also likely to be heterogeneous because of ascertainment differences in referred samples. Our sample comprised referred cases, and thus the higher rate of CD in our cohort may be related to the fact that these children were identified with ADHD and referred. Because this subgroup of children with ADHD + ID are excluded from virtually all clinical, etiologic, and treatment studies, further work is needed to verify our findings. A further limitation of this study is that we were unable to take into account the variable effects of medication timing and dosage. However, in those children receiving a stimulant medication, whether or not they took the medication on the day of testing had no effect on whether they were classified as ADHD + ID or ADHD-only.

A related question that is beyond the scope of the present study is the extent to which the presence of ID in children with ADHD indexes a different etiology. Previous work has shown that children with ADHD + ID are significantly more likely to have large, rare structural deletions or duplications of DNA, called copy number variants (CNVs).^31^ Importantly however, CNVs are also associated with ADHD without ID.^32^ In addition, the presence of such CNVs does not appear to index a distinct pattern of etiologic correlates, in the form of various prenatal and perinatal risk factors, or a distinct clinical profile in children with ADHD with and without ID.^33^ Thus, whether the etiology and risk correlates of ADHD are substantially different in affected children with and without ID remains unclear, and further work is needed to explore this question.

Our results, together with other findings, suggest that excluding children with ADHD from services and interventions on the basis of the presence of mild ID is clinically unwarranted, given that children with ADHD and ID do not seem to differ from those without ID in terms of ADHD subtype and number of ADHD symptoms. They are more likely to have CD, however. It also appears that they differ from children with ID alone, suggesting that ID does not drive the link to conduct problems. Thus, services that deal with ADHD should be well placed to manage ADHD in children with mild ID; however, they will need access to the types of social and clinical interventions that will also help manage associated conduct problems.

## Figures and Tables

**Table III:** Clinical features of children with ADHD + ID (clinical sample) and those with ID only (ALSPAC sample) after matching the samples based on IQ

Variables	ADHD + ID (n = 58)		ID-only (n = 58)		Statistics		
	n (%)	Mean (SD)	n (%)	Mean (SD)	OR	95% CI	*P*
Age, years		10.6 (2.9)		10.8 (0.1)	0.96	0.80-1.15	.64
Male sex	50 (86.2)		31 (53.4)		0.18	0.07-0.46	2.5E-04
ODD symptom count		4.1 (2.3)		0.2 (0.7)	5.08	2.63-9.79	1.2E-06∗
CD symptom count (out of 7)		1.1 (1.5)		0.0 (0.3)	11.41	2.82-46.13	6.4E-04∗
ODD diagnosis	28 (48.3)		2 (3.4)		32.09	6.26-164.56	3.2E-05∗
CD diagnosis	16 (16.7)		0 (0.0)		NA	NA	NA†

∗ A adjusted for the covariates: child's age at time of assessment, sex, and IQ.

† Because there were no CD diagnoses in the ID-only group, statistical calculation was not applicable.

**Table IV:** Clinical features of children with ADHD + ID compared with those with IQ 70-84 and those with IQ ≥85

Variables	Group 2: IQ 70-84 (n = 380)		Statistics∗ (group 2 compared with ADHD + ID)			Group 3: IQ ≥85 (n = 494)		Statistics∗ (group 3 compared with ADHD + ID)		
	n (%)	Mean (SD)	OR	95% CI	*P*†	n (%)	Mean (SD)	OR	95% CI	*P*†
Age, years		10.2 (2.8)	0.87	0.80-0.94	.0004		9.8 (2.7)	0.82	0.76-0.88	4.5E−07
Male sex	317 (83.4)		1.28	0.68-2.44	.45	422 (85.4)		1.10	0.58-2.08	.76
DSM-IV Combined ADHD diagnosis	277 (73.5)		0.54	0.30-0.96	.04	354 (72.0)		0.49	0.28-0.87	.01
DSM-IV Inattentive ADHD diagnosis	21 (5.5)		1.44	0.52-3.99	.49	30 (6.1)		1.79	0.66-4.87	.25
DSM-IV Hyperactive-Impulsive ADHD diagnosis	41 (10.8)		1.86	0.76-4.54	.17	46 (9.3)		1.55	0.63-3.76	.34
DSM-III-R ADHD diagnosis only	34 (9.0)		1.78	0.67-4.69	.25	55 (11.2)		2.23	0.86-5.77	.10
DSM-IV ODD diagnosis	160 (42.6)		1.19	0.75-1.90	.45	239 (48.8)		1.51	0.95-2.37	.08
DSM-IV CD diagnosis	82 (21.6)		0.55	0.34-0.89	.02	54 (10.9)		0.25	0.15-0.41	7.8E−08
ADHD symptoms: inattentive		7.3 (1.7)	0.79	0.46-1.36	.40‡		7.3 (1.7)	0.80	0.47-1.36	.41‡
ADHD symptoms: hyperactive-impulsive		7.8 (1.3)	0.81	0.45-1.45	.48‡		7.7 (1.6)	0.66	0.37-1.18	.16‡
ADHD symptoms: total		15.2 (2.4)	0.94	0.85-1.04	.21		15.1 (2.4)	0.92	0.83-1.01	.09
DSM-IV ODD symptom count		3.9 (2.4)	0.95	0.86-1.05	.33		3.7 (2.3)	0.89	0.81-0.99	.02
DSM-IV CD symptom count		1.3 (1.76)	0.53	0.37-0.75	.0003‡		0.9 (1.3)	0.34	0.24-0.48	1.5E−09‡
DSM-IV anxiety symptoms		1.1 (1.9)	0.84	0.58-1.20	.33‡		1.1 (2.0)	0.82	0.57-1.18	.29‡
DSM-IV depression symptoms		1.2 (1.4)	0.72	0.47-1.11	.13‡		1.3 (1.3)	0.86	0.57-1.31	.49‡

∗ All clinical analyses are adjusted for the covariate child's age.

† Critical *P* value corrected for multiple testing: *P* < .003.

‡ Transformed.

**Table I:** Clinical features of the ADHD + ID and ADHD-only groups

Variable	ADHD + ID (n = 97)		ADHD only (n = 874)		Statistics∗		
	n (%)	Mean (SD)	n (%)	Mean (SD)	OR	95% CI	*P*†
Age, y		11.4 (3.0)		10.0 (2.7)	1.19	1.11-1.28	4.6E-06
Male sex	84 (86.6)		739 (84.6)		0.85	0.46-1.56	.60
DSM-IV Combined ADHD diagnosis	79 (82.3)		631 (72.6)		1.96	1.12-3.40	.02
DSM-IV Inattentive ADHD diagnosis	5 (5.2)		51 (5.8)		0.62	0.23-1.62	.32
DSM-IV Hyperactive-Impulsive ADHD diagnosis	6 (6.2)		87 (10.0)		0.59	0.25-1.41	.23
DSM-III-R ADHD diagnosis only	5 (5.2)		89 (10.2)		0.49	0.19-1.26	.14
DSM-IV ODD diagnosis	36 (37.5)		399 (46.1)		0.74	0.48-1.14	.17
DSM-IV CD diagnosis	35 (36.1)		136 (15.6)		2.69	1.69-4.28	2.8E-05
ADHD symptoms: inattentive		7.5 (1.5)		7.3 (1.7)	1.25	0.75-2.09	.39‡
ADHD symptoms: hyperactive-impulsive		7.8 (1.4)		7.8 (1.5)	1.38	0.79-2.40	.25‡
ADHD symptoms: total		15.4 (2.1)		15.1 (2.4)	1.08	0.98-1.18	.12
DSM-IV ODD symptom count		4.1 (2.3)		3.8 (2.4)	1.09	0.99-1.19	.08
DSM-IV CD symptom count		2.1 (2.2)		1.0 (1.5)	2.38	1.71-3.32	2.5E-07‡
DSM-IV anxiety symptoms		1.2 (2.0)		1.1 (1.9)	1.21	0.86-1.70	.28‡
DSM-IV depression symptoms		1.8 (1.8)		1.3 (1.3)	1.26	0.84-1.89	.26‡

∗ All clinical analyses were adjusted for the covariate child's age.

† Critical *P* value corrected for multiple testing: *P* < .003.

‡ Transformed.

**Table II:** Clinical features of children with ADHD + ID (clinical sample) and those with ID only (ALSPAC sample)

Variables	ADHD + ID (n = 97)		ID-only (n = 58)		Statistics		
	n (%)	Mean (SD)	n (%)	Mean (SD)	OR	95% CI	*P*
Age, y		11.4 (3.0)		10.8 (0.1)	1.10	0.96-1.27	.16
Male sex	84 (86.6)		31 (53.4)		0.18	0.08-0.39	1.4E-05
ODD symptom count		4.1 (2.3)		0.2 (0.7)	5.54	2.86-10.75	4.0E-07∗
CD symptom count (out of 7)		1.2 (1.4)		0.0 (0.3)	13.66	3.25-57.42	3.6E-04∗
ODD diagnosis	47 (49.0)		2 (3.4)		30.99	6.38-150.39	2.0E-05∗
CD diagnosis	16 (16.7)		0 (0.0)		NA	NA	NA†

*NA*, not applicable.

∗ All clinical analyses adjusted for the covariates child's age at time of assessment, sex, and IQ.

† Because there were no CD diagnoses in the ID-only group, statistical calculation was not applicable.
